# Re-examination of the contribution of substrates to energy expenditure during high-intensity intermittent exercise in endurance athletes

**DOI:** 10.7717/peerj.3769

**Published:** 2017-09-06

**Authors:** Zübeyde Aslankeser, Şükrü Serdar Balcı

**Affiliations:** Faculty of Sports Science, Selçuk University, Konya, Turkey

**Keywords:** Substrate oxidation, High-intensity intermittent exercise, Endurance athlete

## Abstract

**Background:**

It has been believed that the contribution of fat oxidation to total energy expenditure is becoming negligible at higher exercise intensities (about 85% VO_2max_). The aim of the present study was to examine the changes in substrate oxidation during high-intensity interval exercise in young adult men.

**Methods:**

A total of 18 healthy well-trained (aged 19.60 ± 0.54 years, BMI = 22.19 ± 0.64 kg/m^2^, *n* = 10) and untrained (aged 20.25 ± 0.41 years, BMI = 22.78 ± 0.38 kg/m^2^, *n* = 8) young men volunteered to participate in this study. After an overnight fast, subjects were tested on a cycle ergometer and completed six 4-min bouts of cycling (at ∼80% VO_2max_) with 2 min of rests between intervals. Energy expenditure and the substrate oxidation rate were measured during the experiment by using indirect calorimetry. The blood lactate concentration was collected immediately after each interval workout.

**Results:**

The fat oxidation rate during each workout was significantly different between the untrained and the athlete groups (*p* < 0.05), and the carbohydrate (CHO) oxidation rate during the experiment was similar between groups (*p* > 0.05). Moreover, lactate concentration significantly increased in the untrained group (*p* < 0.05), whereas it did not significantly change in the athlete group during the workouts (*p* > 0.05). Fat contribution to energy expenditure was significantly higher in the athlete group (∼25%) than in the untrained group (∼2%).

**Conclusions:**

The present study indicates that 17 times more fat oxidation was measured in the athlete group compared to the untrained group. However, the athletes had the same CHO oxidation rate as the recreationally active subjects during high-intensity intermittent exercise. Higher fat oxidation rate despite the same CHO oxidation rate may be related to higher performance in the trained group.

## Introduction

The main sources of energy for skeletal muscles are carbohydrate (CHO) and fat. While CHO is utilized in aerobic and anaerobic systems, fat is utilized only in the aerobic system (McArdle, Katch & Katch, 2010). The preference of the substrate depends mainly on the exercise intensity (Achten & Jeukendrup, 2003). CHO oxidation increases as the exercise intensity increases. Exercise at an intensity greater than 65% of VO_2max_ rapidly decreases fat oxidation (Achten & Jeukendrup, 2004; Van Loon et al., 2001; Venables, Achten & Jeukendrup, 2005). Although fatty acids are oxidized as the main source of energy during low-intensity exercise, mobilization of fatty acids decreases when exercise intensity increases (Hodgetts et al., 1991; Sidossis et al., 1997). There have been many studies focusing on why fat oxidation decreases during high intensity exercise and some possibilities have been suggested concerning this issue (Romijn et al., 1993; Sidossis et al., 1997; Van Loon et al., 2001). The first possibility is that the mobilization of fatty acids to mitochondria decreases. It was noted that the activity of carnitine palmitoyltransferase (CPT), which is responsible for the transfer of fatty acids to mitochondria, decreases during high-intensity exercise, and sufficient CHOs in the cell lead to a decrease in fat oxidation (Ockner, 1990). While the activity of the circulatory system increases during exercise, lipolysis decreases because adipose tissue blood flow decreases (Hodgetts et al., 1991; Romijn et al., 1995). Moreover, the oxidation of fatty acids decreases even though there are sufficient fatty acids in the blood during moderate- and high-intensity exercise. Glycogen breakdown and CHO oxidation are the main sources of energy, particularly at 85% of VO_2max_. In this situation, lactate increase in muscle and blood (Coyle, 1995) is reported to lead to decrease in fat mobilization (Maughan & Gleeson, 2010). Fats contain more energy when compared to CHOs, and CHOs are limited energy resources during long-term exercise (Hawley, Brouns & Jeukendrup, 1998), for this reason, fats provide more energy than CHO (McArdle, Katch & Katch, 2010).

Previous studies focused mainly on low- and moderate-intensity exercises. In addition, there are fewer studies related to the contributions of high-intensity interval training to the rate of substrate oxidation and energy expenditure in endurance athletes. Stepto et al. (2001) reported that elite cyclists could sustain aerobic power outputs during high-intensity (86% VO_2max_) intermittent (8  × 5-min) exercise. Researchers associated this ability with high rates of glycogenolysis and total energy expenditure in the athletes. However, fat oxidation rates of the athletes increased during the last set of the intervals. Moreover, we think that the increase in the fat oxidation rate, which was ignored by the researchers, contributed to sustainability of the exercise. In contrast, Coggan et al. (1995) found that plasma glucose concentration of the endurance-trained cyclists increased meaningfully when compared to untrained subjects during 30 min high-intensity (80% VO_2max_) exercise. Researchers stated that hyperglycaemia that was observed in trained subjects was related to the rate of glucose utilization instead of high glucose production rate. Low rate of glucose utilization suggests that fat is oxidized to a great extent, even during high-intensity exercise. It is typically known that fats are predominantly oxidized at low intensities and carbohydrates are predominantly oxidized at high intensities. Nevertheless, Hetlelid et al. (2015) highlighted that elite runners have three times more fat oxidation than non-elite runners during high-intensity interval workouts, and thus the contribution of fat oxidation to high-intensity interval exercise should be monitored. However, authors in that study took into consideration the fat oxidation rate during exercise and rest periods, for this reason their results did not show substrate oxidation rates only during high intensity interval exercise. In their study, the RER values have increased rapidly at rest between intervals. It could cause a decrease in the fat oxidation rate due to the nonmetabolic CO_2_ increases (Romijn et al., 1992).

Changes in substrate oxidation and differences between endurance athletes and untrained subjects were not studied in detail. Therefore, the changes in substrate oxidation during high-intensity intervals in endurance athletes were examined in this study. The hypothesis of this study was that the contribution of fat oxidation increases energy expenditure because endurance-trained athletes have adaptations that enhance the fat oxidation rate in high-intensity exercise.

## Methods

### Participants

Eighteen young adult males (10 well-trained cyclists competing at national and international levels and 8 physical education students who had not experienced any endurance training before) voluntarily participated in the present study. The physical characteristics of the participants are shown in Table 1. The study was approved by the Ethics Committee of the Faculty of Sports Science at Selçuk University. The procedures and risks were thoroughly explained to each participant and a written informed consent was obtained prior to participation in the study.

**Table 1:** Physical characteristics of the participants.

Variables	Untrained (*n* = 8)	Athlete (*n* = 10)
	Mean ± SD	Mean ± SD
Age (years)	20.25 ± 1.16	19.60 ± 1.71
Body weight (kg)	69.39 ± 4.43	69.89 ± 6.64
Body height (cm)	174.50 ± 5.08	177.50 ± 6.04
Body mass index (kg/m^2^)	22.78 ± 1.06	22.19 ± 2.03
VO_2max_ (ml/kg/min)	47.77 ± 5.00	60.85 ± 2.83^*^
Body fat (%)	13.24 ± 1.55	11.79 ± 2.43
Lactate at rest (mmol/l)	1.44 ± 0.47	1.79 ± 0.75

**Notes.**

* Significantly different from the untrained group (unpaired *t*-test, *p* < 0.05).

### General design

The participants came to the laboratory on three separate days for the following purposes: (1) anthropometric measurements and a familiarization test; (2) the cycle test for the determination of the maximal oxygen uptake; (3) the high-intensity interval test consisting of 6 × 4-min bouts of cycling at ∼82% VO_2max_ for athletes and at ∼76% VO_2max_ for untrained group, separated by 2 min of recovery. Furthermore, blood lactate concentration was measured immediately after each interval workout. The participants arrived at the laboratory after a 12-hour fast. They were instructed to avoid caffeine, alcohol, strenuous and exhaustive physical activity for two days before the exercise tests.

All participants were familiar with cycle exercise and high-intensity exercise workouts. The exercise tests were separated by at least four days and never more than seven days. Each subject was tested at the same time of the day (09:00–11:00 a.m.) to minimize the effects of diurnal biological variation.

### Experimental design

Subjects were barefoot and in their underwear for the anthropometric measurements. Weight was measured with a Seca scale (Seca 711, Deutschland), and height was measured with a stadiometer that was incorporated into the scale. The body mass index (BMI) was calculated for all participants. Body fat estimations were made using the four-site (biceps, triceps, subscapular and suprailiac) skinfold technique according to the method of Durnin & Womersley (1974). Skinfold measurements were made on the right side of the body using a Holtain caliper. The same technique was performed during all anthropometric measurements for all subjects.

All participants were familiarized with the cycle ergometer (Monark 839 E, Sweden) before the beginning of the experiment. Subjects performed an exercise test until exhaustion on the cycle ergometer, and expired gases were collected via facemask and analyzed (Cosmed K4b^2^ portable metabolic system, Cosmed S.R.L., Rome, Italy) for determination of VO_2max_. The subjects started pedaling at 50 W (for the sedentary group) or 100 W (for the athlete group). The pedaling cadence for each subject was set at 70 revolutions/min (rpm). Subjects cycled during a 3-min warm-up period at the 50-W workload, after which the workload was increased by 25 W/min every 2 min until exhaustion or the predetermined exclusion criteria were met. The criteria for achieving VO_2max_ were as follows: no increase in VO_2_ (plateau) for a given increase in workload, maximum heart rate with respect to age (220 beats min^−1^ minus age), a VE/VO_2_ value close to 30 L min^−1^ and a respiratory exchange ratio (RER) greater than 1.15. Calculations were performed for the average oxygen uptake over the last 60 s of the test.

The Monark cycle ergometer was used for the high-intensity interval test. The exercise test consisted of a 5-min warm-up period at a workload chosen by each subject. At the end of the warm-up period, subjects performed an interval test consisting of six work periods which lasted 4-min at 70–100 rpm and separated by 2-min rest periods. During the rest periods, the subjects rested by sitting on the cycle ergometer. VO_2_, VCO_2_ and HR were recorded during the exercise and cool down periods. The workload was adjusted during the test to ensure that the average corresponding VO_2_ during exercise was equated to ∼80% VO_2max_. Capillary blood was collected from a fingertip immediately after each work period to quantify lactate concentration (Nova Biomedical Lactate Plus Blood Lactate Analyser, Waltham, MA).

### Indirect calorimetry and calculations

Breath-by-breath measurements were collected throughout exercise tests using an indirect calorimeter (Cosmed K4B^2^, Italy). The indirect calorimetry system was calibrated prior to each test according to manufacturer’s specifications. During the interval exercise test, average values for VO_2_ and VCO_2_ were calculated for each workout. Substrate oxidation during the interval exercise test was calculated according to the following stoichiometric equations (Frayn, 1983), assuming that the urinary nitrogen excretion rate was negligible:

\begin{eqnarray*}& & \text{CHO oxidation}(\mathrm{g}\;{\mathrm{min}}^{-1})=4.55\times {\mathrm{V CO}}_{2}(\mathrm{l}\;{\mathrm{min}}^{-1})-3.21\times {\mathrm{V O}}_{2}(\mathrm{l}\;{\mathrm{min}}^{-1}) \end{eqnarray*}

\begin{eqnarray*}& & \text{Fat oxidation}(\mathrm{g}\;{\mathrm{min}}^{-1})=1.67\times {\mathrm{V O}}_{2}(\mathrm{l}\;{\mathrm{min}}^{-1})-1.67\times {\mathrm{V CO}}_{2}(\mathrm{l}\;{\mathrm{min}}^{-1}). \end{eqnarray*}

If RER values were above 1, the fat oxidation was accepted as zero and data were calculated as such.

The correction equation was used for the calculation of energy expenditure assuming a negligible contribution of protein oxidation (Jeukendrup & Wallis, 2005).

\begin{eqnarray*}\text{Energy expenditure}(\mathrm{kcal}\;{\mathrm{min}}^{-1})=0.550\times {\mathrm{V CO}}_{2}(\mathrm{l}\;{\mathrm{min}}^{-1})+4.471\times {\mathrm{V O}}_{2}(\mathrm{l}\;{\mathrm{min}}^{-1}). \end{eqnarray*}

The relative contributions of fat and CHO oxidation to energy expenditure were calculated using the following equations (McGilvery & Goldstein, 1983):

\begin{eqnarray*}& & \text{% Fat}=[(1-\text{RER})/0.29]\times 100 \end{eqnarray*}

\begin{eqnarray*}& & \text{%}\;\mathrm{CHO}=[(\mathrm{RER}-0.71)/0.29]\times 100. \end{eqnarray*}

### Statistical analyses

Statistical analyses were performed using SPSS version 16.0. A two-way (split-plot) ANOVA with repeated measures was used to test the effect of high-intensity interval exercise on substrate oxidation. The two factors were group (untrained and athletes) and repeated measures (six high-intensity interval exercise bouts). When repeated measures effect was significant via split-plot ANOVA, one-way repeated measures analysis of variance with the post hoc Bonferroni test was applied to identify whether the interval exercise was responsible for the difference. When group effect was significant in the split-plot ANOVA, unpaired t-tests were used to compare mean values between groups. Statistical significance was set at a level of *p* < 0.05, and data were expressed as the means ± standard deviation.

## Results

Age, body weight, height, body mass index and body fat percentage were similar in both groups (*p* > 0.05). The maximal oxygen uptake was significantly higher in athletes than in the untrained group (*p* < 0.05).

As shown in Table 2, oxygen uptake during each interval workout was significantly higher in the athlete group than in the untrained group (group effect; *F* = 28.97, *p* < 0.05). However, changes in oxygen uptake during high-intensity exercise workouts were similar in both groups (workout effect; *F* = 2.48, group-workout interaction effect; *F* = 3.35, *p* > 0.05). The respiratory exchange ratio (RER) was significantly higher in the untrained group than in the athlete group (group effect; *F* = 25.09, *p* < 0.05). RER decreased in both untrained (*p* < 0.05) and athlete groups during the workouts (workout effect; *F* = 6.32). Nevertheless, changes in RER during workouts were not different between the groups (group-workout interaction effect; *F* = 1.37, *p* > 0.05).

**Table 2:** The substrate oxidation rates of the untrained and athlete groups during the high intensity interval exercise.

		Untrained (*n* = 8)	Athlete (*n* = 10)	ANOVA		
Variables	Workouts	Mean ± SD	Mean ± SD	W	G	G × W
VO_2_ (ml)	1st	2271.56 ± 344.92	3470.54 ± 403.17^¥^			
	2nd	2506.56 ± 406.92	3530.60 ± 477.12^¥^			
	3rd	2549.42 ± 431.60	3590.85 ± 522.20^¥^	2.48	28.97^*^	3.35
	4th	2640.88 ± 487.36	3437.28 ± 2790.20^¥^			
	5th	2638.36 ± 509.34	3444.14 ± 294.25^¥^			
	6th	2618.57 ± 425.71	3442.25 ± 268.30^¥^			
VCO_2_ (ml)	1st	2480.63 ± 425.65	3317.32 ± 437.16^¥^			
	2nd	2585.04 ± 432.96	3239.07 ± 408.83^¥^			
	3rd	2575.37 ± 430.24	3328.70 ± 404.72^¥^	0.14	15.37^*^	1.65
	4th	2727.10 ± 566.56	3174.26 ± 236.43^¥^			
	5th	2667.49 ± 530.09	3163.00 ± 252.30^¥^			
	6th	2635.84 ± 413.36	3185.55 ± 281.87^¥^			
RER	1st	^acf^1.09 ± 0.06	0.96 ± 0.05^¥^			
	2nd	^b^1.03 ± 0.03	0.92 ± 0.04^¥^			
	3rd	^ca^1.01 ± 0.04	0.93 ± 0.05^¥^	6.32^*^	25.09^*^	1.37
	4th	^d^1.03 ± 0.04	0.93 ± 0.08^¥^			
	5th	^e^1.01 ± 0.04	0.92 ± 0.07^¥^			
	6th	^fa^1.01 ± 0.02	0.93 ± 0.07^¥^			
Fatox (g/min)	1st	0.01 ± 0.04	0.29 ± 0.27^¥^			
	2nd	0.01 ± 0.04	0.49 ± 0.24^¥^			
	3rd	0.05 ± 0.10	0.46 ± 0.31^¥^	1.38	15.66^*^	0.99
	4th	0.03 ± 0.05	0.47 ± 0.40^¥^			
	5th	0.05 ± 0.08	0.49 ± 0.40^¥^			
	6th	0.03 ± 0.06	0.45 ± 0.38^¥^			
CHOox (g/min)	1st	4.00 ± 0.95	3.95 ± 0.99			
	2nd	3.72 ± 0.73	3.40 ± 0.60			
	3rd	3.53 ± 0.70	3.62 ± 0.68	1.33	0.48	0.51
	4th	3.93 ± 1.08	3.41 ± 1.02			
	5th	3.67 ± 0.86	3.34 ± 0.98			
	6th	3.59 ± 0.57	3.44 ± 1.06			
EE (kcal/min)	1st	11.52 ± 1.77	17.34 ± 2.02^¥^			
	2nd	12.63 ± 2.05	17.57 ± 2.35^¥^			
	3rd	12.81 ± 2.16	17.89 ± 2.54^¥^	1.98	27.84^*^	3.14
	4th	13.31 ± 2.49	17.11 ± 1.31^¥^			
	5th	13.26 ± 2.56	17.14 ± 1.40^¥^			
	6th	13.16 ± 2.13	17.14 ± 1.29^¥^			
Fatox contribution to EE (%)	1st	1.24 ± 3.50	17.14 ± 15.80^¥^			
	2nd	0.95 ± 2.68	28.17 ± 12.14^¥^			
	3rd	3.81 ± 7.35	25.24 ± 14.43^¥^	1.27	17.34^*^	0,89
	4th	2.16 ± 4.03	27.11 ± 22.86^¥^			
	5th	3.78 ± 6.35	28.49 ± 21.67^¥^			
	6th	1.97 ± 3.76	26.75 ± 21.75^¥^			
CHOox contribution to EE (%)	1st	98.76 ± 3.50	82.86 ± 15.80^¥^			
	2nd	99.05 ± 2.68	71.83 ± 12.14^¥^			
	3rd	96.19 ± 7.35	74.76 ± 14.43^¥^	1.27	17.34^*^	0,89
	4th	97.84 ± 4.03	72.89 ± 22.86^¥^			
	5th	96.22 ± 6.35	71.51 ± 21.67^¥^			
	6th	98.03 ± 3.76	73.25 ± 21.75^¥^			
HR (beats/min)	1st	^abcdef^157.14 ± 8.52	^acdef^154.77 ± 15.53			
	2nd	^bacf^171.10 ± 10.09	^b^160.79 ± 14.22			
	3rd	^cabf^174.84 ± 9.37	^ca^168.55 ± 17.97	35.61^*^	1.47	1.14
	4th	^da^178.98 ± 10.95	^daf^169.39 ± 15.03			
	5th	^ea^178.84 ± 11.56	^eaf^171.30 ± 15.94			
	6th	^fabc^180.54 ± 9.39	^fade^173.42 ± 15.26			
Lactate (mmol/l)	1st	^abcdef^5.84 ± 1.81	3.36 ± 0.76^¥^			
	2nd	^ba^7.79 ± 1.92	3.70 ± 1.13^¥^			
	3rd	^caf^8.40 ± 2.35	3.95 ± 1.46^¥^	8.45^*^	34.52^*^	3.70^*^
	4th	^da^9.04 ± 2.64	4.10 ± 1.21^¥^			
	5th	^ea^9.20 ± 2.66	3.69 ± 1.39^¥^			
	6th	^fac^9.08 ± 2.66	4.36 ± 2.03^¥^			

**Notes.**

TITLE W workout effect G group effect G × W group-workout interaction effect

* Significant main and/or interaction effect (split-plot ANOVA with repeated measures, *p* < 0.05).

^a–f^ Similar superscripts in the same column indicate significant differences (repeated one-way ANOVA with Bonferroni, *p* < 0.05).

¥ Significantly different from the untrained group for measurement time points (unpaired *t*-test, *p* < 0.05).

**Figure 1:**
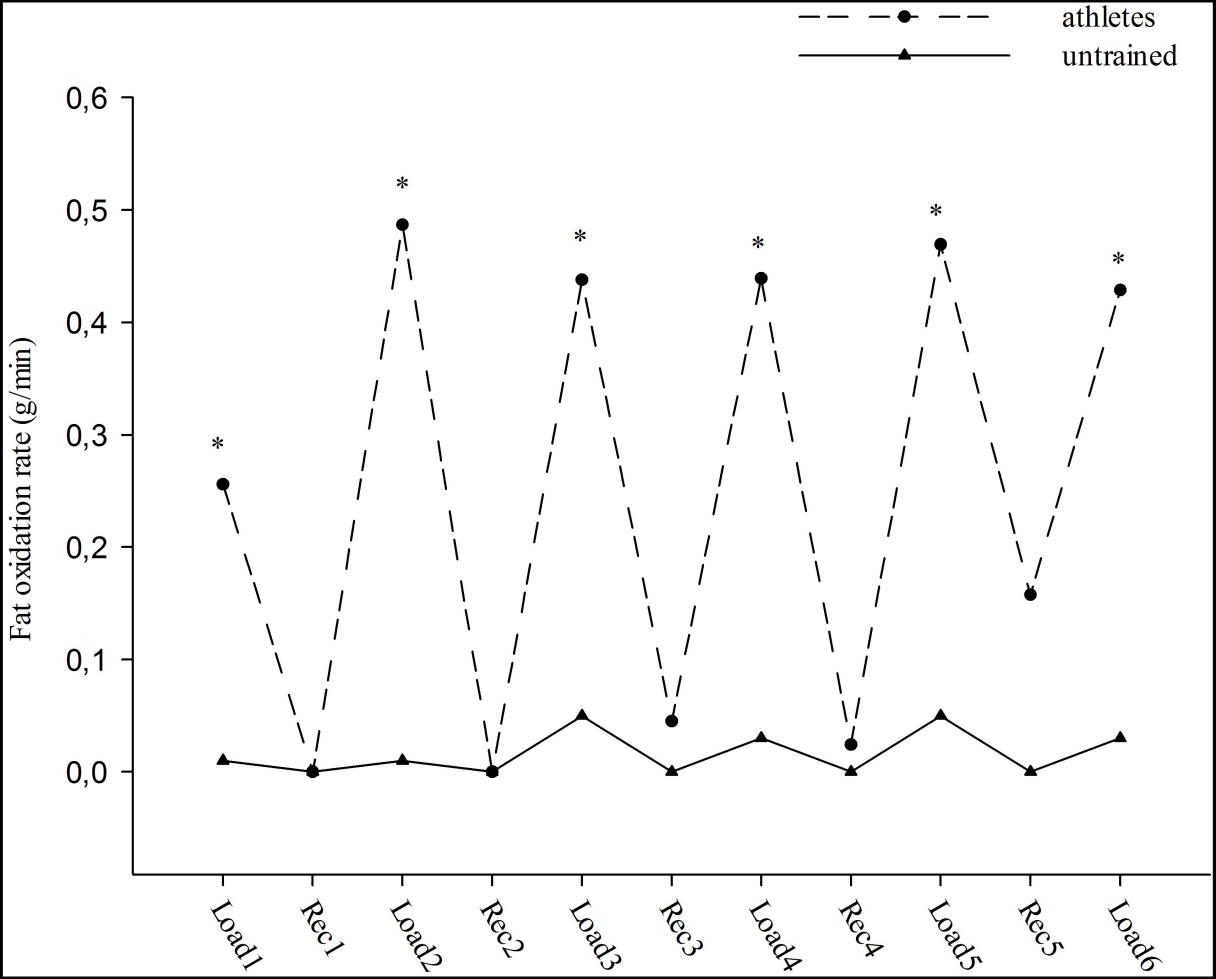
The fat oxidation rate during high intensity intermittent exercise. Load: Workouts, Rec: Recovery. ^∗^*p* < 0.05: significant differences between athletes and untrained groups.

**Figure 2:**
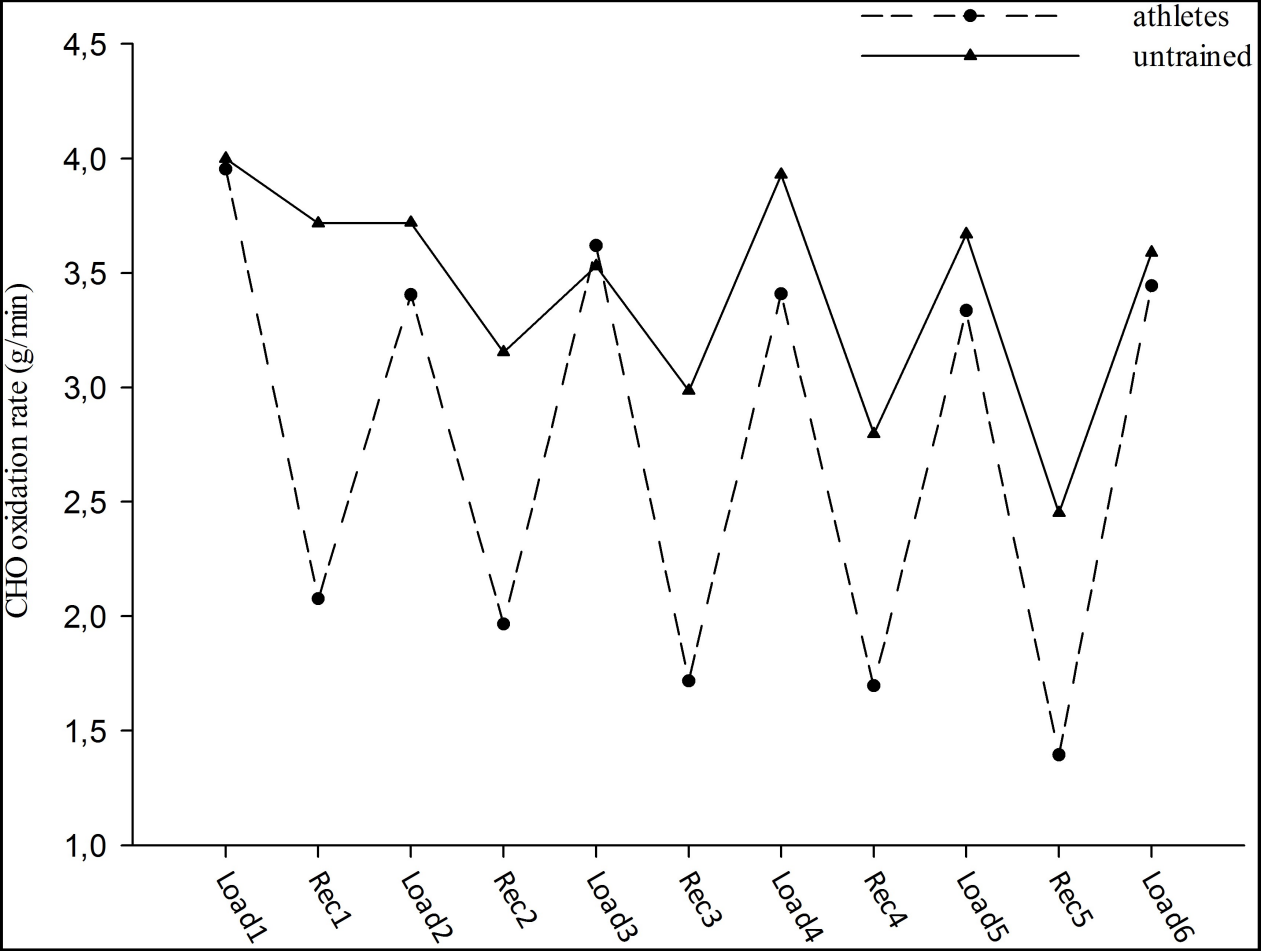
CHO oxidation rate during high intensity intermittent exercise. Load: Workout, Rec: Recovery.

Fat oxidation rates for each workout were significantly different between both groups (*p* < 0.05), (0.03 ± 0.02 g/min and 0.44 ± 0.11 g/min, respectively). The athletes had significantly higher fat oxidation rates than untrained subjects at each interval workout during the test (approximately seventeen-fold) (group effect; *F* = 15.66, *p* < 0.05). Moreover, the changes in fat oxidation rates during the interval workouts were similar in the groups (workout effect; *F* = 1.38, group-workout interaction effect; *F* = 0.99, *p* > 0.05), (Fig. 1). In contrast, no significant main effect of workouts (*F* = 1.33) and groups (*F* = 0.48) was observed for CHO oxidation rates. In addition, no significant interaction effect was observed between groups and workouts (*F* = 0.51) for CHO oxidation rates (*p* > 0.05). CHO oxidation rates at each interval workout and the changes in oxidation rates were similar for both groups (3.74 ± 0.29 g/min and 3.53 ± 0.28 g/min, respectively), (Fig. 2).

Energy expenditure during the interval workouts was higher in the athlete group than in the untrained group (group effect; *F* = 27.84, *p* < 0.05), and the percent of fat and CHO contribution to energy expenditure was significantly different between the groups (group effect; *F* = 17.34, *p* < 0.05). The fat contribution to energy expenditure was significantly higher in the athlete group (∼25%) than in the untrained group (∼2%), and the CHO contribution was significantly lower in the athlete group compared to the untrained group (∼75% and ∼98%, respectively) (*p* < 0.05). In addition, energy expenditure (workout effect; *F* = 1.98, group-workout interaction effect; *F* = 3.14), percent of fat and CHO contribution to energy expenditure (work out effect; *F* = 1.27, group-workout interaction effect; *F* = 0.89) did not significantly change during the six workouts for both groups (*p* > 0.05).

The effect of interval workouts on heart rate was significant (*F* = 35.61, *p* < 0.05). Additionally, heart rate significantly increased in both groups during the workouts (*p* < 0.05). However, heart rate during the workouts were not different between the groups (group effect; *F* = 1.47). The increase in heart rate during the workouts was similar in the untrained and athlete groups (group-workout interaction effect; *F* = 1.14), (*p* > 0.05). Moreover, lactate concentration was significantly higher in the untrained group than in the athlete group during high-intensity interval exercise (group effect; *F* = 34.52, *p* < 0.05). Changes in lactate concentration during the high-intensity exercise workouts were significantly different between the untrained and the athlete groups (workout effect; *F* = 8.45, group-workout interaction effect; *F* = 3.70, *p* < 0.05), (8.23 ± 0.83 mmol/L and 3.86 ± 0.42 mmol/L, respectively). Lactate concentration significantly increased in the untrained group (*p* < 0.05), whereas it did not significantly change in the athlete group during the workouts (*p* > 0.05).

## Discussion

The major finding in this study, in which substrate oxidation rates were examined during high-intensity interval exercise, was that 17 times more fat oxidation took place in the athlete group compared to the untrained group although both groups had similar CHO oxidation rates. The contribution of fat to energy expenditure was 25% and 2% in athletes and untrained subjects, respectively, and the CHO contribution to energy expenditure was 75% and 98% for athletes and active subjects, respectively.

Although the upper limit of lipolysis in humans is not known, more fat mobilization occurs in subjects with a high aerobic capacity. Endurance athletes have a higher lipolytic rate than untrained subjects at the same exercise intensity (Martin et al., 1993; Klein et al., 1996; Coggan et al., 2000; Lima-Silva et al., 2010). When substrate oxidation rates are examined during exercise, fats are known to be oxidized at low intensity, and CHOs are oxidized mostly at high intensity (Romijn et al., 1993). For this reason, the contribution of fat oxidation to total energy expenditure during exercise above 85% VO_2max_ is generally ignored (Achten & Jeukendrup, 2003). González-Haro et al. (2007) observed that the rate of fat oxidation was at minimum at 88% of VO_2max_ in competitive endurance athletes. However, in our study, it was observed that some adaptations led to increased fat oxidation rates and the contribution of fats to energy expenditure even during high-intensity exercise. In this study, a high fat oxidation rate (approximately 0.44 ± 0.11 g/min) in athletes during high-intensity interval exercise was maintained at all loads. Likewise, Hetlelid et al. (2015) reported that endurance athletes had three times more fat oxidation than the untrained group, and 33% of the total energy expenditure was supplied by fats in well-trained athletes during a running exercise that included intervals with a similar intensity to our study. However, researchers in that study calculated the substrate oxidation rate even during the rest periods between exercise bouts. Therefore, findings related to the contribution of fat to total energy expenditure may be higher than in the present study. Moreover, it is notable that the rate of the contribution of fat to total energy expenditure was over 25% during high-intensity exercise in endurance athletes.

The adaptations that increase fat oxidation after endurance training can be explained with the increase in triglyceride density and an increase in the quantity of mitochondrial enzymes and activation (Goodpaster et al., 2001; Tarnopolsky et al., 2007). It was demonstrated that such factors as increases in the capillarization of muscles, carnitine transferase that enhances fatty acid mobilization, fatty acid-binding protein (Molé, Oscai & Holloszy, 1971) and catecholamines (Stallknecht et al., 1995) influenced the increase in fat oxidation. Regular endurance training boosts the fat oxidation rate by facilitating changes at a molecular level. It is noted that fat oxidation may increase independently from the number of mitochondria because of the increase in free fatty acids (FFA) that follow a high-fat diet (Dyck et al., 1996). Tunstall et al. (2002) found an increase in the mobilization of fatty acids and gene expression, which supported the transport of fatty acids from plasma and the mitochondrial membrane even after 9-day moderate-intensity endurance training. In this study, the cyclists who formed the athlete group had trained for more than six years. We think that the above-mentioned adaptations caused an increase in the fat oxidation rate even at the high-intensity workload because athletes had trained at moderate- and high-intensity levels over a long period of time. Furthermore, Westgarth-Taylor et al. (1997) reported decreased CHO oxidation and increased fat oxidation during incremental tests between 60–80% VO_2max_ after 6-week interval training using a similar interval exercise protocol as in this study (6–9 × 5 min at 85–88% VO_2peak_). Also, high intensity interval training increased fatty acid- binding protein content in total muscle plasma membrane (Talanian et al., 2007). Trapp, Chisholm & Boutcher (2007) noted that it would be suitable to include high-intensity interval exercise into training programs in order for fat loss. Stepto et al. (2001) stated that endurance athletes retained power outputs during exercise comprised of 8 × 5-min bouts at an intensity of 86% of VO_2max_ with 1 min rest between bouts. They reported an increase in the fat oxidation rate and no change in the CHO oxidation rate; however, they completely ignored the contribution of fat oxidation in their study.

In studies where indirect calorimetry was used, when the exercise intensity increased, VO_2_ directly showed the amount of oxygen consumption of the muscle, and VCO_2_ was higher than that produced by the cell. In this stage, where activity of the anaerobic energy system increased, an extra increase of CO_2_ is seen because the bicarbonate buffer system is activated, which may cause overestimation of CHO oxidation and underestimation of fat oxidation rate (Achten & Jeukendrup, 2004). Also, some studies have suggested a direct effect of lactate ions on fat oxidation (Boyd et al., 1974; Fredholm, 1971). Indeed, because exercise intensity was high enough to increase lactate concentration in the capillary blood, it is clearly observed that CHOs were anaerobically metabolized. For these reasons, fat oxidation rate in athletes was 17 times higher than untrained groups because of the high lactate levels and nonmetabolic CO_2_ production. Also, metabolic adaptations at cellular levels in athlete group cause more fat oxidation at high intensity intervals. Taking into account all of these factors, fat oxidation rate in athletes was recorded 17 times higher than nonathletes. Similar CHO oxidation rates were observed in the athlete and untrained groups during all workloads. Nevertheless, the contribution of CHOs to the total energy expenditure was approximately 75% in athletes compared to 98% in the untrained group. The percentage difference of evaluated energy expenditure confirms that athletes effectively utilize fats at high intensities, even though CHO oxidation rates were similar. It was observed that lactate concentrations in the athletes did not change throughout all workloads (approximately 3.86 ± 0.42 mmol/L). Despite continuous exercise, lactate steady-state was a result of a high aerobic capacity in the athletes. Lactate is metabolized by the liver, muscles and other tissues. The fact that there is a balance between lactate production and lactate elimination shows that intervals are carried out aerobically (Tschakert & Hofmann, 2013; Billat, 2001). Muscles that are active during the exercise play an important role in lactate elimination (Stanley et al., 1986). Mitochondria increase in volume and density as a result of chronic workloads and provide basic mechanisms for the elimination of lactate (Thomas et al., 2004). It was stated that after chronic endurance training, capillarization increases (Denis et al., 1986), lactate carrier proteins increase (Dubouchaud et al., 2000) and lactate elimination occurs faster due to the above-stated reasons. It was observed that lactate concentration increased rapidly in the untrained group after the first workload, and lactate levels were higher than those of the athletes during all workloads. The fact that more lactate production occurred in the untrained group despite similar CHO oxidation rates between the two groups shows that glucose was mostly metabolized anaerobically. Moreover, it is known that the increase in acidity is a result of anaerobic metabolism in the muscle, which suppresses oxidative metabolism. Lactate accumulation during exercise is acutely related to a decrease in fat oxidation (Achten & Jeukendrup, 2004; Bircher & Knechtle, 2004; González-Haro et al., 2007). Besides, Peric et al. (2016) have suggested a high correlation between anaerobic threshold and minimum fat oxidation. In this study, the lactate levels of all participants may be explained with this relationship. This finding provides another explanation for the difference in the contribution of fat oxidation and fat oxidation rate to energy expenditure.

The relative exercise intensities were not the same between the groups. It was ∼82% in the athletes and ∼76% in the untrained group. The athletes were accustomed to high intensity interval exercise, but it was very difficult for the untrained subjects because of their lack of exercise performance. While the untrained subjects had lower exercise intensity during workouts than athletes, the HR and lactate levels in untrained group were higher than the athletes. Although the athletes had a bit higher relative exercise intensity than untrained subjects during the interval exercise test, the higher fat oxidation rates during high intensity interval exercise in athletes were very important. Although exercise intensity for the athlete group was measured as 80% VO_2max_ and higher, the fact that the RER was under 1 and fat oxidation was high may suggest a constraint in the measurement of VO_2max_. However, we began the test with different resistances in the VO_2max_ protocol for the athletes and the untrained subjects as explained in the methods section. There are many studies stating that a high-fat diet increases fat oxidation during exercise (Burke & Hawley, 2002; Burke et al., 2000; Lambert et al., 2001; Yeo et al., 2011). In this study, the last meal was consumed 12 h before the interval exercise. Participants were instructed to prepare meals that were comprised of 60% carbohydrate, 20% fat and 10% protein; however, the participants were not monitored.

Endurance athletes and road cyclists participating in this study use high-intensity workloads. Hour-long training sessions are not at the same intensity throughout the session and include intervals with incline and speed changes (Hawley et al., 1997; Jeukendrup, Craig & Hawley, 2000). High fat oxidation during competition is important in terms of retarding depletion of the CHO stores and preventing a decrease in performance (Volek, Noakes & Phinney, 2015). In our study, higher VO_2_ and lower lactate concentration in the athletes during high- intensity interval exercise partially explains the difference in fat oxidation rates between the groups. 17 times more fat oxidation in the athlete group despite the same CHO oxidation rate as recreationally active individuals shows that athletes can perform these intervals via aerobic pathway due to a developed aerobic capacity, and the athletes are capable of adaptations that can facilitate continuous fat oxidation even during high-intensity exercise. Accordingly, further studies should be conducted in terms of the contribution of fats to total energy expenditure in athletes during high-intensity exercise.

##  Supplemental Information

10.7717/peerj.3769/supp-1 Supplemental Information 1 Raw data calculations

10.7717/peerj.3769/supp-2 Supplemental Information 2 Data set

10.7717/peerj.3769/supp-3 Supplemental Information 3 Results of statistics

## Additional Information and Declarations

### Data Availability

The following information was supplied regarding data availability: The raw data has been supplied as a Supplemental File.
